# Expression of Tiam1 predicts lymph node metastasis and poor survival of lung adenocarcinoma patients

**DOI:** 10.1186/1746-1596-9-69

**Published:** 2014-03-24

**Authors:** Shuguang Liu, Yumei Li, Wenjuan Qi, Yunfei Zhao, Aili Huang, Wenjie Sheng, Bin Lei, Peixin Lin, Haili Zhu, Wenxia Li, Hong Shen

**Affiliations:** 1 Department of Pathology, Nanfang Hospital, Southern Medical University, 510515 Tonghe, Guangzhou, People’s Republic of China; 2 Department of Pathology, School of Basic Medical Sciences, Southern Medical University, 510515 Tonghe, Guangzhou, People’s Republic of China

**Keywords:** Lung adenocarcinoma, Lymph node metastases, Immunohistochemistry, Tiam1

## Abstract

**Background:**

To assess the value of Tiam1 in predicting lymph node metastasis and survival after curative resection in patients with lung adenocarcinoma.

**Methods:**

Immunohistochemical staining for Tiam1 was performed on 98 adenocarcinoma and 30 normal lung tissues. The association of Tiam1 protein expression with the clinicopathological characteristics and the prognosis of lung adenocarcinoma were subsequently assessed.

**Results:**

Immunohistochemical analysis showed that 60 of 98 (61.22%) adenocarcinoma tissues showed high expression of Tiam1, and high Tiam1 expression was significantly associated with advanced TNM stage (P < 0.0005) and lymph node status (P < 0.0005) of lung adenocarcinoma. Moreover, the lung adenocarcinoma patients with low Tiam1 expression had higher overall survival than patients with high Tiam1 expression (log rank value = 10.805, P = 0.001). High expression of Tiam1 predicted poor overall survival of patients in stages I + II (P = 0.006). Furthermore, multivariate analysis indicated that high expression of Tiam1 protein was an independent prognostic factor for overall survival (P = 0.011) in patients with lung adenocarcinoma.

**Conclusion:**

These findings suggest for the first time that Tiam1 expression may be beneficial in predicting lymph node metastasis and survival of patients with lung adenocarcinoma. A future study will investigate whether Tiam1 can serve as a novel therapeutic target in lung adenocarcinoma.

**Virtual slides:**

The virtual slide(s) for this article can be found here: http://www.diagnosticpathology.diagnomx.eu/vs/1377798917111123.

## Background

Lung cancer is the leading cause of cancer deaths worldwide. Adenocarcinoma is the most frequent type of lung cancer, which has a tendency to spread to the lymph nodes [1]. Currently, although therapy strategies have improved, the prognosis of lung adenocarcinoma patients is still poor. The main reason is that many patients have high frequency of metastasis at diagnosis. Identification of molecular targets that contribute to adenocarcinoma metastasis can help in selecting the best treatment strategy and improve lung adenocarcinoma outcomes.

Tiam1, also called T-cell lymphoma invasion and metastasis-inducing factor, was first identified as a metastasis-related gene in the aggressive mice T lymphoma cells [2]. It is a member of guanine nucleotide exchange factors (GEFs) and regulates the guanosine triphosphatase to facilitate the exchange of guanosine diphosphate for guanosine triphosphate. This indicates that Tiam1 is involved many biological processes by regulating a variety of GTP-binding proteins. It has been reported that Tiam1 participates in cytoskeleton rearrangement, and cell migration and mobility in T-lymphoma cells, fibroblasts and epithelial cells [3-6]. Accumulating evidences have shown that Tiam1 expression plays a role in cancer progression and metastasis by activating Rho-like GTPases and Tiam1-Rac1 pathway in various cancers, such as nasopharyngeal carcinoma [7], breast cancer [8], colorectal cancer [9], retinoblastoma [10], hepatocellular carcinoma [11] and Ras-induced skin tumors [12]. Moreover, many studies have shown that Tiam1 expression might be a new and independent predictor of prognosis in various solid tumors [13-15]. However, the potential prognostic relevance of Tiam1 expression in lung adenocarcinoma has no been investigated.

In this study, we attempted to investigate the expression of Tiam1 in lung adenocarcinoma using immunohistochemical staining and identify its relationship to lymph node metastasis, prognosis and clinicopathological features.

## Materials and methods

### Patients and tissue samples

Formalin-fixed, paraffin-embedded samples of 98 lung adenocarcinoma tissue and 30 normal lung tissues were obtained from surgical patients between 2002 and 2006 from NanFang hospital of Southern Medical University, Guangzhou, China. Of 98 patients with lung adenocarcinoma, there were 53 men and 45 women, between the ages of 34 and 79 years (median, 57 years). The average survival time was 37.7 months for these patients, and ranged from 1 to 101 months. Lung adenocarcinomas were graded and staged according to 2009 WHO/IASLC [16]. Diagnosis was confirmed by the Department of Pathology of Nan Fang hospital. No patients in this study had received any adjuvant systemic therapy before surgery. The Ethics Committee of Southern Medical University gave prior approval for this study, and every patient provided written informed consent.

### Immunohistochemistry (IHC)

Immunohistochemical study of Tiam1 was performed on formalin-fixed, paraffin-embedded, 5 μm-thick sections using Envision method. In brief, the sections were deparaffinized and dehydrated. Endogenous peroxidase activity was halted through the administration of 0.3% hydrogen peroxidase for 10 min. After having been rinsed in phosphate-buffered saline (PBS), the tissue sections were processed in a 0.01 M citrate buffer (PH6.0) and treated with high-pressure antigen retrieval. The sections were incubated with primary polyclonal antibody rabbit anti-Tiam1 (1: 50; Santa Cruz Biotech, USA) overnight at 4°C. Followed by washing in PBS, the slides were subsequently incubated with Polymer Helper (PV9000 kits, Zhongshan Bio Corp., Beijing, China) for 20 min. Then, after washing again with PBS, the sections were incubated with poly peroxidase-goat-anti-rabbit IgG antibody (PV9000 kits, Zhongshan Bio Corp., Beijing, China) for 30 min. The results were visualized with diaminobenzidine (Zhongshan Bio Corp., Beijing, China) at room temperature for 7 min. Finally, the sections were counterstained with hematoxylin, dehydrated, cleared, and mounted. The sections not incubated with primary antibody served as negative controls. The sections with confirmed positive expression of Tiam1 were used as a positive control, and only cytoplasmic staining was considered positive.

### Evaluation of immunohistochemical staining

For the assessment, representative areas of each section were selected, and cells were counted in five fields at 400-fold magnification. Scores for the percentage of tumor cells stained positive were as follows: 0, <5%; 1, ≥5 to 25%; 2, >25 to 50%; 3, >50 to 75%; and 4, >75%. Staining intensity was scored as 0, lack of staining; 1, mild staining; 2, moderate staining; and 3, strong staining. Based on the semi-quantitative score calculated by multiplying these two values (which ranged from 0-12), the stained sections were defined as either low expression(0–3) or high expression(≥4). The immunostained slides were assessed by two independent pathologists, who were blinded to the clinicopathologic information.

### Statistical analysis

Statistical analyses were carried out using the SPSS 13.0 statistical software package. Pearson’s chi-square (×2) test was used to analyze the association between Tiam1 expression and each clinicopathological parameter. The survival curves were generated according to the Kaplan–Meier method, and differences in survival were analyzed by log-rank test. Survival data were evaluated using univariate and multivariate Cox regression analyses. P value less than 0.05 was considered statistically significant.

## Result

**Figure 1:**
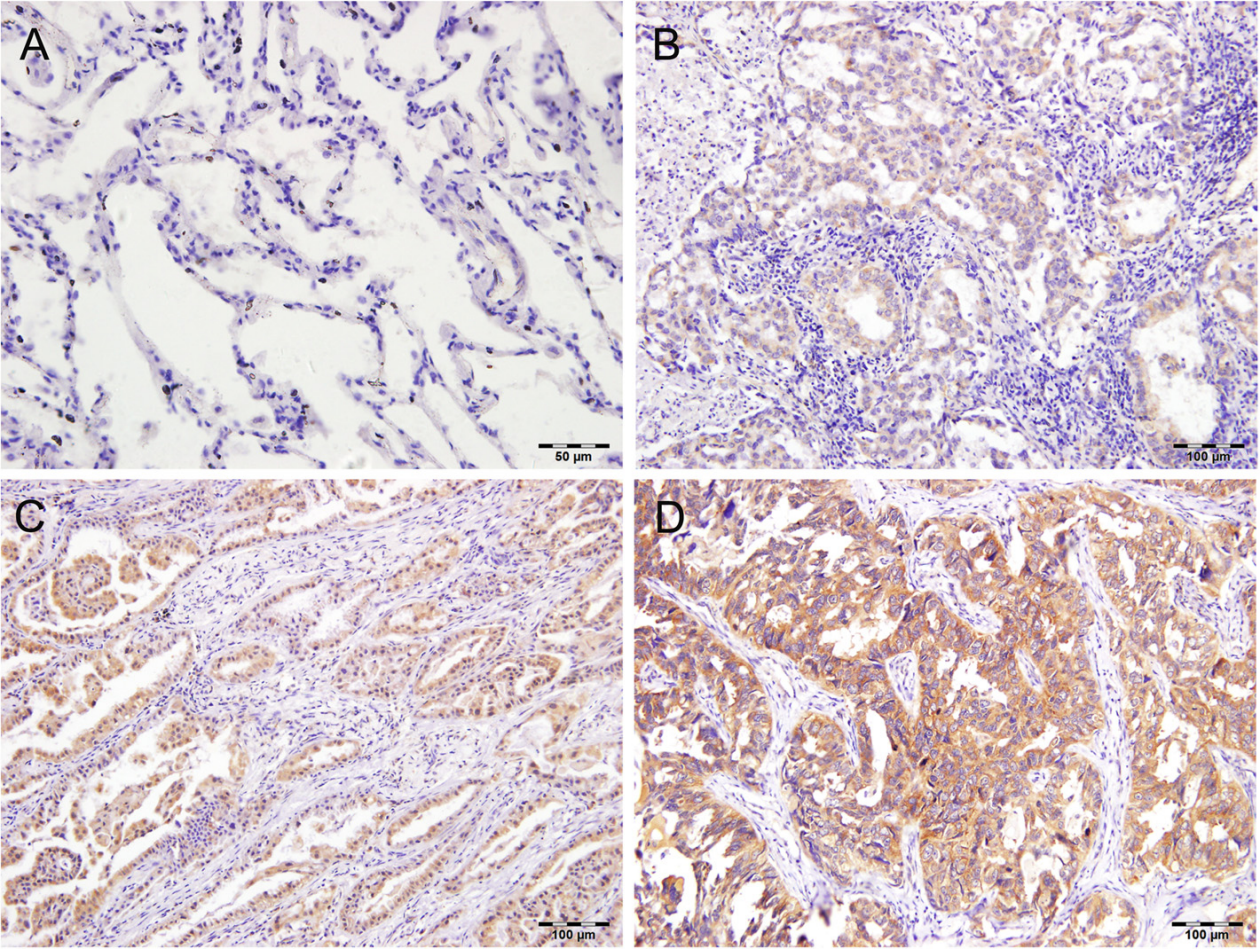
**Expression of Tiam1 in lung adenocarcinoma and normal lung tissue. A** Negative expression in normal lung tissue; **B** Mild expression in lung adenocarcinoma tissue; **C** Moderate expression in lung adenocarcinoma tissue; **D** Strong expression in lung adenocarcinoma tissue.

**Figure 2:**
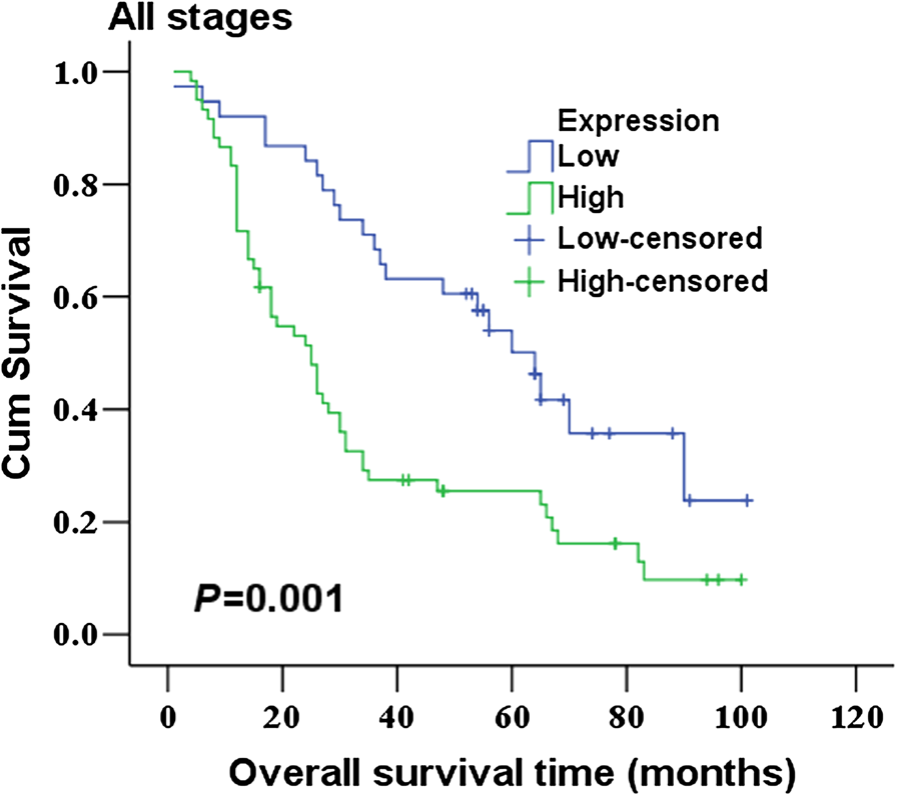
**Kaplan–Meier curves of overall survival defined by Tiam1 expression.** Patients with low Tiam1 expression had higher overall survival than patients with high Tiam1 expression (*P* = 0.001, log rank test).

**Figure 3:**
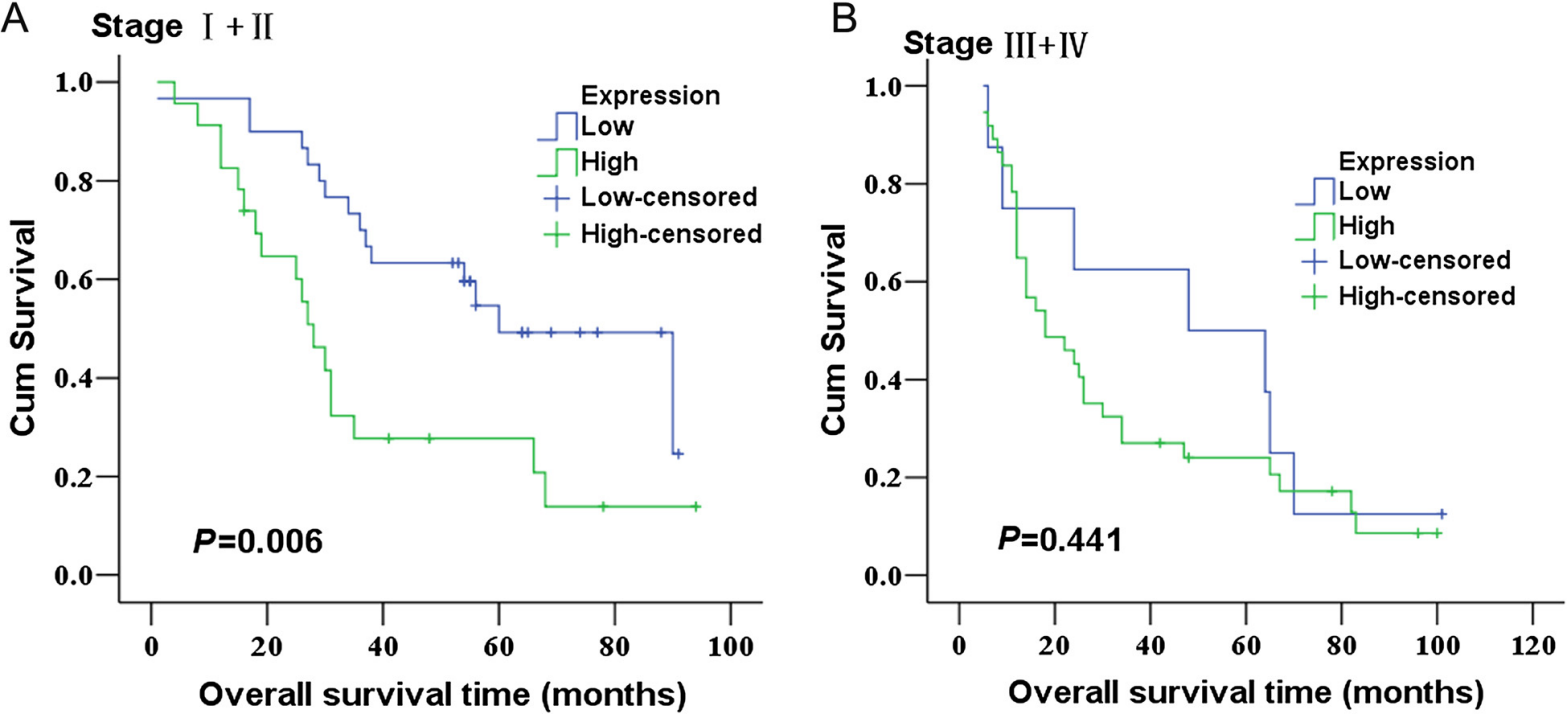
**Association of Tiam1 expression with overall survival of patients with lung adenocarcinoma in different stages.** High expression of Tiam1 predicted poor overall survival in stages I + II (P = 0.006) but not stages III + IV (P = 0.441) patients (Figure 3**A**, **B**).

Tiam1 protein expression level was determined by immunohistochemistry in 98 lung adenocarcinoma tissues and 30 non-neoplastic tissues (used as a normal control). As shown in Figure 1, There was no protein expression or very weak staining found in normal lung epithelia. Conversely, the immunoreactive patterns of Tiam1 were predominantly positively identified in the cancer tissues. Tiam1 protein was stained clearly brown-yellow and localized in the cell cytoplasm. Of these tumor tissues, sixty exhibited high expression (61.22%), and 38 low expression (38.78%). The correlation between Tiam1 protein expression and clinicopathological features was listed in Table 1. Tiam1 protein expression was significantly associated with TNM stage (P < 0.0005) and lymph node status (P < 0.0005), but not with patients’ age, gender, differentiation and tumor size. Overall survival analysis using the Kaplan–Meier method showed that patients with low Tiam1 expression had higher overall survival than patients with high Tiam1 expression (Figure 2; log rank value = 10.805, P = 0.001). Moreover, expression of Tiam1 predicted poor overall survival in stages I + II (P = 0.006) but not stages III + IV (P = 0.441) patients (Figure 3A, B). Upon the univariate analysis with the cox proportional hazards model, TNM stage (P = 0.013), lymph node status (P = 0.015) and Tiam1 expression (P = 0.002) were associated with overall survival. Multivariate analyses revealed that high expression of Tiam1 was an independent predictor of an unfavorable prognosis (Hazard ratio, 2.085; 95% CI, 1.186-3.667; P = 0.011; Table 2).

**Table 1:** Association of Tiam1 expression with clinicopathological characteristics from lung adenocarcinoma

**Characteristics**	**Cases**	**Low expression**	**High expression**	***P***
		**n (%)**	**n (%)**	
Age (years)				
<60	55	18(33)	37(67)	0.165
≥60	43	20(47)	23(53)	
Gender				
Male	53	24 (45)	29 (55)	0.151
Female	45	14 (31)	31 (69)	
Differentiation				
High	39	14 (36)	25 (64)	0.634
Low	59	24 (41)	35 (59)	
Tumor size (cm)				
≤3	42	19 (45)	23 (55)	0.255
>3	56	19 (34)	37 (66)	
TNM stage				
I	27	19 (70)	8 (30)	<0.0005
II	26	11 (42)	15 (58)	
III	39	7 (18)	32 (82)	
IV	6	1 (17)	5 (83)	
Lymph node metastasis				
No	39	24 (62)	15 (38)	<0.0005
Yes	59	14 (24)	45 (76)	

**Table 2:** Univariate and multivariate prognostic analysis for lung adenocarcinoma patients

**Variables**	**Univariate**			**Multivariate**		
	**HR**	**95% CI**	***P***	**HR**	**95% CI**	***P***
Sex	0.971	0.609-1.549	0.903	0.912	0.566-1.468	0.704
Age	0.920	0.578-1.467	0.727	1.068	0.662-1.724	0.788
Tumor size	1.545	0.956-2.496	0.076	1.247	0.734-2.119	0.415
Differentiation	1.339	0.824-2.178	0.239	1.238	0.732-2.095	0.426
TNM stage	1.811	1.136-2.887	0.013	1.028	0.526-2.011	0.935
Lymph node status	1.842	1.125-3.015	0.015	1.300	0.669-2.529	0.439
Tim1 expression	2.268	1.367-3.764	0.002	2.085	1.186-3.667	0.011

Note: 95% CI means 95% confidence interval, HR means Hazard ratio.

## Discussion

Lung adenocarcinoma, a major subtype of non-small-cell lung carcinomas (NSCLC), is one of the most deadly human carcinomas, accounting for approximately one third of all lung cancer cases. Distinguished from lung squamous cell carcinoma, lung adenocarcinoma has its unique biological and clinical characteristics, such as frequent mutations of epidermal growth factor receptor and anaplastic lymphoma kinase. Despite advances in diagnosis and compositive therapy, the overall survival rate for patients with lung adenocarcinoma is still low. The main reason of cancer-related mortality is lymph node metastasis. Therefore, prediction of regional lymph node status and patient survival is important, because it can influence the choice of treatment strategies.

Tiam1 was originally identified a metastasis-related gene of T lymphoma. As one of the guanine nucleotide exchange factors (GEFs), it was crucially involved in a variety of tumor signaling pathway through the regulation of Rho GTPases functions. HOU et al. found that down-regulation of Tiam1 using RNA interference resulted in inhibition of in vitro invasiveness in giant-cell lung carcinoma cells [17]. Liu et al. reported that Tiam1 gene plays an important role in the proliferation, invasion, and metastasis of colorectal cancer cells [18]. Tiam1 expression was suggested to be closely associated with motility in human breast cancer cell lines and was necessary to maintain the motile phenotype [19]. Studies also showed that up-regulation of Tiam1 was associated with metastasis of hepatocellular carcinoma [20] and gastric cancer [21]. These data indicated that Tiam1 expression can induce invasion and metastasis of tumor cells. And more notably, high expression of Tiam1 in tumor cells, implying a poor prognosis, has been observed in several solid tumors. For example, high Tiam1 expression is an independent predictor of decreased disease-free survival for patients with prostate cancer [13]. Overexpression of Tiam1 correlates with poor prognosis in hepatocellular carcinoma [22]. Recently, Du et al. has suggested that high Tiam1 expression is associated with poor overall survival in patients with primary gallbladder carcinoma [23]. Nevertheless, whether these finding of Tiam1 can be extended to lung adenocarcinoma remains elusive.

In this study, we analyzed the expression of Tiam1 in 98 cases of lung adenocarcinoma tissues using immunohistochemistry. We found that Tiam1 expression was upregulated in lung adenocarcinoma compared to normal lung tissues, which suggest that Tiam1 like other members of the GEFs family, has an oncogenic role in the tumorigenesis of lung adenocarcinoma. Our finding is in consistence with previous studies that Tiam1 expression was found in multiple different cancer tissues, confirms a significant relation between Tiam1 expression and genesis and development of lung adenocarcinoma.

Our analysis further showed that Tiam1 overexpression correlates with lymph node metastasis of patients with lung adenocarcinoma, which suggests that Tiam1 might play an important role in the progression and invasion of lung adenocarcinoma. This result strongly supports our previous observation that Tiam1 expression was closely associated with 1ymph node metastasis in NSCLC [24]. In addition, evidence also suggested that expression of Tiam1 is closely associated with lymph node metastasis in other tumor tissues [25,26]. Molecularly, Tiam1 participates in cytoskeleton reorganization, cell adhesion and cell migration. Thus, we anticipate that Tiam1 may reshape lung cancer cells to make them easier to spread to the lymph nodes or enhance reciprocity between tumor and stroma, though the precise molecular mechanism of Tiam1’s action in lung adenocarcinoma remains to be clarified. These results indicate that Tiam1 expression in lung adenocarcinoma may be predictive of lymph node metastasis. With this approach, patients at high risk of lymph node metastasis could be identified for more aggressive treatment.

In the current study, we are the first to report the prognostic value of Tiam1 expression for patients with lung adenocarcinoma. According to Kaplan–Meier survival analysis, Tiam1 protein expression was found to be inversely correlated with patient’s overall survival. Increased expression of Tiam1 protein was significant predictor of poor prognosis for patients with lung adenocarcinoma, especially for patients with stage I-II cancer. Multivariate analysis revealed a significant negative relationship between the Tiam1 overexpression and overall survival. Therefore, we can conclude that Tiam1 serves as a biomarker for predicting prognosis of lung adenocarcinoma patients. Since Tiam1 overexpression can be a new predictor of poor prognosis of patients in a variety of tumors, it may be served as a new and independent predictor of prognosis for patients with lung adenocarcinoma as well. Yet, due to our limited sample size, with only six patients of stage IV, the observed association of Tiam1 expression with pathological stage should be verified by further studies.

In summary, expression of Tiam1 protein was significantly higher in lung adenocarcinoma tissue than in normal tissue. Higher Tiam1 expression is associated with lymph node metastasis, and is also an independent prognostic marker of poor survival in patients with lung adenocarcinoma. Tiam1 may serve as a useful molecular marker for lung adenocarcinoma progression and invasion. However, further studies are needed to verify our current findings, and we will investigate whether Tiam1 is a useful therapeutic target in lung adenocarcinoma.

## Competing interests

The authors declare that they have no competing interests.

## Authors’ contributions

HS, SGL and YML participated in the design of the study. HS and SGL wrote the manuscript. YFZ, ALH, WJS and BL carried out the H&E and IHC staining. WJQ, HLZ and PXL collected the clinical data and reviewed H&E and IHC slides. WXL performed the statistical analysis. All authors read and approved the final manuscript.
